# Transfer of elements from soil to earthworms and ground beetles in boreal forest

**DOI:** 10.1007/s00411-023-01027-2

**Published:** 2023-04-17

**Authors:** Soroush Majlesi, Päivi Roivainen, Anne Kasurinen, Tiina Tuovinen, Jukka Juutilainen

**Affiliations:** Department of Environmental and Biological Sciences, University of Eastern Finland, P.O. Box 1627, 70211 Kuopio, Finland

**Keywords:** Radioecology, Ecotoxicology, Concentration ratio, Bioaccumulation, Field study, Mesocosm study

## Abstract

Data on the transfer of elements (such as heavy metals) and their radionuclides into organisms is needed for assessing environmental risks. The current data on many elements, species and environments is limited, but more information can be obtained both from field studies and experimental laboratory studies. However, it is essential to evaluate whether experimental studies adequately predict transfer in natural conditions. Moreover, because of the sparsity of species-specific empirical data, it is a common practice in current radioecological modelling to use data available for related species under the assumption that transfer into organisms is similar within broader taxonomic groups. Earthworms and ground beetles are examples of important invertebrates living near soil surface in terrestrial ecosystems. In this study, the transfer of 34 elements from soil to these organisms was studied in a field study conducted in boreal forest. The earthworm concentrations were compared to the values obtained in an experimental mesocosm study using soil from the field site and were found to be highly correlated. This indicates that the results of mesocosm studies can be used for predicting the transfer of elements from soil to fauna in natural conditions. Furthermore, concentrations in individual earthworm and beetle species were found to be similar to those observed in broader groups of related species, indicating that the generic approach used in current radioecological models may be useful for predicting uptake of elements into single species.

**Supplementary Information:**

The online version contains supplementary material available at 10.1007/s00411-023-01027-2.

## Introduction

Data on the transfer of elements (such as heavy metals) and their radionuclides in the environment is needed in both ecotoxicology and radioecology for assessing the risks associated with, e.g., mining activities, accidental contamination, and disposal of radioactive and other wastes. However, empirical data on transfer of elements into many wildlife species is limited (Mann et al. 2011; Copplestone et al. 2013; IAEA 2014; Brown et al. 2016; Ali et al. 2019). Soil organisms feeding on detritus and/or other soil animals have an important role in transferring elements in forests (Haygarth and Jarvis 2002; De Vries and Groenenberg 2009; Ishii et al. 2017; Mortensen et al. 2018). Moreover, any contamination in such organisms may result in transfer of elements to higher trophic levels such as birds and small mammals.

Earthworms (*Annelida: Oligochaeta*) and ground beetles (*Coleoptera: Carabidae*) are important organisms in terrestrial ecosystems. Earthworms are ecosystem engineers and have crucial role in nutrient cycling and shaping of soil structure (Blouin et al. 2013; Medina-Sauza et al. 2019). Earthworms spend most of their time in the litter layer (epigeic species), in the topsoil (endogeic species) or deep in the soil (anecic species), (Lavelle 1988). They mostly feed on organic material in various stages of decay and are prey of many other organisms (Lavelle 1988; Curry and Schmidt 2007). Ground beetles spend most of their time on soil surface and on the litter layer (Jelaska et al. 2007). They have an important role in soil trophic webs and most of them prey on many organisms including earthworms (Jelaska et al. 2007; Butovsky 2011; Ikeda et al. 2012).

Concentration ratios (CR) and the analogous bioaccumulation factors and bioconcentration factors are widely used in models to predict uptake of radionuclides and metals into biota (McGeer et al. 2003; IAEA 2014). CR is defined as the ratio of element/radionuclide concentration in biota to the corresponding concentration in media (IAEA 2010, 2014). It is generally assumed that the transfer of stable and radioactive isotopes of the same element is similar and data on stable isotopes can therefore be used in models predicting transfer of radionuclides (IAEA 2010), although this assumption has been questioned (Wood et al. 2013; Beresford et al. 2020) and researchers should be aware of the potential pitfalls. The databases presenting CRs are mainly compiled from the results of field studies, but experimental datasets in artificial micro- or mesocosms are also used for this purpose. However, for combining the evidence from experimental and field studies, it is essential to know whether meso- and microcosm studies adequately predict uptake of elements in natural conditions.

In this study, the transfer of 34 elements from soil to earthworms and ground beetles was investigated at a boreal forest site under field conditions. The aim of the study was to provide CR values needed in transfer models. The second aim was to compare the earthworm data of the field study with data from an experimental mesocosm study, previously carried out using soil collected from the same forest site (Tuovinen et al. 2016a). Because it is challenging to obtain species-specific empirical data for all species, it is a common practice in current radioecological modelling to predict transfer into organisms using generic categories such as ‘’arthropods’’ or ‘’annelids’’ rather than a single genus or species (IAEA 2014). This approach is based on the assumption that uptake into an organism can be approximated using data available for a related species. The third aim was to evaluate the validity of this assumption in earthworms and ground beetles.

## Materials and methods

### Sampling site

**Fig. 1:**
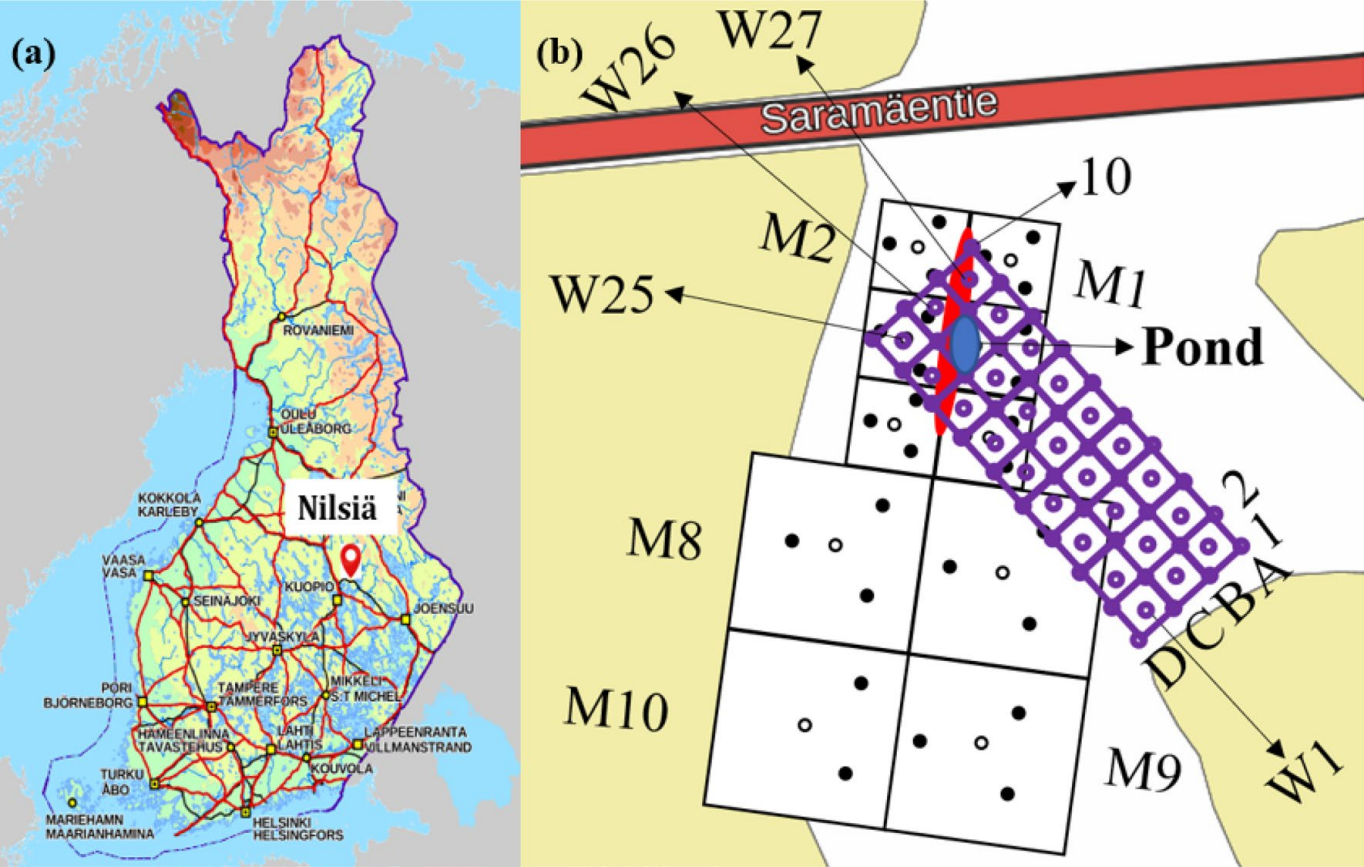
**a** The location of sampling site in Nilsiä, Eastern Finland. **b** A grid of 10 squares marked from M1 to M10, each including three pitfall traps for beetles (black circles in triangle-shaped arrangements) around the excavation pit (red) in 2007 (except for M10 with two traps since it was partly located outside the forest) and a grid of 27 squares including 40 pitfall traps for beetles (marked from A1 to D10, purple circles) on the corners of the squares in 2008. Earthworms were sampled from the center of each square in 2007 (marked with black circles with no fill from M1 to M10) and 2008 (sampling points marked from W1 to W27, no-fill purple circles) by digging. The map is adapted from National Land Survey of Finland, license CC 4.0 (Topographic map raster 1:50,000; 08/2021)

The field study was conducted in Nilsiä, Eastern Finland, at a forest site (N63° 04′ and E 27° 54′ WGS84) where small-scale uranium (U) ore prospecting was carried out in the 1960s (Fig. 1a). Sampling was mostly implemented in the area around the old uranium excavation pit, with a small pond at one end (Fig. 1b). A more detailed description of the study site can be found in Roivainen et al. (2011a, b). Samples were collected between June and August both in 2007 and 2008.

### Field sampling

The beetles were collected using pitfall traps (Barber 1931; Gongalsky 2003). In 2007 the traps were placed on 29 systematically selected sampling points. A grid consisting of 10 squares was established to assist in the sampling point selection (Fig. 1b). Six squares (size 40 m × 40 m) were around the old ore excavation pit (within 30 m from the pit) and four squares (size 60 m × 60 m) in the untouched area (from 40 to 100 m from the pit). There were three sampling points in each square in triangular forms. For the square M10 (located furthest away from the pit) only two points were selected, since it was partly located outside the forest. In 2008, there was a grid of 27 squares (size 10 m × 10 m) around the excavation pit to collect more samples especially from the area of higher U concentrations in soil. A trap was set up to each corner of the squares, totaling of 40 sampling points. Both years, the traps consisted of 200 ml plastic cups covered by a white or red plastic lid to avoid rain and larger animals from entering the cups. Cups contained car engine coolant (50% ethylene glycol solution) to preserve the samples (Holopainen 1992; Lemieux 1999; Koivula et al 2003; Schmidt et al. 2006). The traps were emptied once a week. Beetle species were identified by Nikon SMZ800 microscope (Tokyo, Japan) and frozen at − 20 °C. Before the analysis of element concentration, the thawed samples were dried at 60 °C for 48 h. Because the number of individuals collected at each sampling point was low, samples had to be pooled to obtain enough material for the chemical analyses (Table 1). The main criterion in pooling was the location of traps: samples from traps close to each other were pooled. The number of collected individuals in 2007 also facilitated using species as the secondary criterion in pooling. There were 24 pooled samples in 2007 and five in 2008.

**Table 1:** Pooled sampling points for beetles (n = 24 in 2007 and n = 5 in 2008) and earthworms (n = 2 in 2007 and n = 7 in 2008)

Year	Beetle pooled sampling points (species)	Earthworm pooled sampling points (species)
2007	M1 (*C. caraboides*); M2 (*P. niger, P. melanarius*); M3 (*P. niger)*; M5 (2), (*P. niger, P. melanarius)*; M6 (*P. niger)*; M7 (3), (*P. niger)*; M8 (*P. niger)*; M9 (2), (*P. niger, P. melanarius)*; M10 (5), (*P. niger)*; M1 + M3 (*P. niger, P. melanarius)*; M4 + M6 (*C. caraboides*); M7 + M9 (*P. oblongopunctatus*); M4 + M10 (*P. niger, P. melanarius)*; M8 + M10 (2), (*P. niger*, P. oblongopunctatus*)*; M7 + M8 + M9 + M10 (*C. glabratus*)	M2 (*Lumbricus terrestris, Dendrobaena octaedra*) M4 (*Lumbricus terrestris, Dendrobaena octaedra)*
2008	1 + 2 (*P. strennus, P. oblongopunctatus, P. melanarius, P. niger, C. caraboides, H. quadripunctatus*); 3AB + 4AB + 5AB + 6AB (*P. strennus, P. oblongopunctatus, C. caraboides, H. quadripunctatus, L. termitus*); 3CD + 4CD (*P. strennus, P. oblongopunctatus, P. melanarius, P. niger, C. glabratus, C. caraboides*); 7AB + 8AB + 9AB + 10AB (*P. strennus, P. oblongopunctatus, P. melanarius, P. niger, L. termitus, H. quadripunctatus*); 5CD + 6CD + 8CD + 9D + 10CD (*P. strennus, P. oblongopunctatus, P. melanarius, L. termitus, H. quadripunctatus, C. caraboides*)	W1 (*Aporrectodea caliginosa, Lumbricus terrestris)* W2 + W3 + W5 + W6 (*Aporrectodea caliginosa, Lumbricus terrestris, Lumbricus rubellus*) W4 + W7 (*Aporrectodea caliginosa, Lumbricus terrestris)* W8 + W9 + W11 + W12 + W14 (*Aporrectodea caliginosa, Lumbricus terrestris, Lumbricus rubellus, Lumbricus castaneus)* W10 + W16 + W19 + W22 (*Aporrectodea caliginosa, Lumbricus terrestris, Lumbricus rubellus)* W18 (*Lumbricus terrestris)* W21 + W24 + W27 (*Aporrectodea caliginosa, Lumbricus terrestris, Lumbricus rubellus*,* Lumbricus castaneus*)

Number of samples and the species sampled are given in parentheses

Earthworms were collected around the center of each square by digging. There were two sampling days each year. After collection, the worms were kept in a petri dish at room temperature for 48 h to remove their gut contents. Then they were identified by Nikon SMZ800 microscope (Tokyo, Japan), preserved in 70% ethanol and frozen at − 20 °C. The thawed samples were dried at 60 °C for 48 h before chemical analysis. Similarly to beetles, earthworms collected at several sampling points had to be pooled and location of the sampling points was the main criterion in pooling. The earthworms were pooled into two samples in 2007 and seven samples in 2008.

Soil samples (to a depth of 100 mm within an area of 100 mm × 100 mm) were collected with a spade at each beetle and earthworm sampling point. The moisture content (dry/fresh weight ratio for 24 h at 105 °C), organic matter content (3 h at 550 °C) and pH of the soil samples were on average 21.0% (7.00–35.0%), 13.1% (2.70–36.9%) and 4.40 (4.00–5.10), respectively. The samples were oven-dried (40 °C) for seven days and sieved to ≤ 2 mm for chemical analyses.

### Mesocosm study

The mesocosm study was carried out at the Research Garden of University of Eastern Finland between years 2011 and 2012. The details and full results of the study focusing on studying transfer of Co, Mo, Ni, Pb, Th, U and Zn in boreal food chain were reported by Tuovinen et al. (2016a). In this paper, we report the concentrations of all measured 34 elements in soil and earthworms and compare them to the results of the field study. Mesocosms including soil collected from the field site (near the old excavation pit), plants, earthworms and snails were established in July 2011 and maintained until September 2012. The original study included also mesocosms containing control soil from a forest site near the Research Garden of University of Eastern Finland (N62° 53′ and E27° 37′) but the results of those mesocosms are not reported in this paper. 25 l of the U-rich soil was added to 30 l of plastic container (n = 9) and birches, grasses and ferns were planted on soil. One week after the establishment of the mesocosms, six adult earthworms (*Lumbricus terrestris*) were added to each mesocosm. The earthworms were purchased from a commercial supplier (Yorkshire-Worms, UK). In October 2011, after the growing season, soil and animal samples were collected from six mesocosms for elemental analysis. However, samples from three mesocosms had to be pooled to obtain enough material for analysis. Therefore, two soil and earthworm samples were analyzed. The three remaining mesocosms were over-wintered in a dark cold room with gradual decease of temperature (from + 8 to + 1) from November 2011 until May 2012. The mesocosms were then returned to the greenhouse and new individuals of earthworms (n = 10 per mesocosm) were added to the system in July 2012 to guarantee sufficient number of animals for the second sampling. Soil (n = 3) and earthworm (n = 3) samples were collected in September 2012 for element analysis. During the growing season, the temperature of the mesocosms was ± 20 ℃ under light regime of 18 h light and 6 h dark.

### Chemical analysis

Element concentrations in animal and soil samples were analyzed after nitric acid digestion in a microwave oven (procedure following US-EPA standard 3051). The samples from the field study were measured by inductively coupled plasma-mass spectroscopy (Perkin Elmer Sciex Elan 5000) or inductively coupled plasma-atomic emission spectroscopy (Thermo Jarrel Ash Iris Advantage) in the laboratory of Labtium Ltd. in Espoo, Finland. The elemental analysis of the samples from the mesocosm study was carried out by inductively coupled plasma-mass spectroscopy (Perkin Elmer Sciex Elan 6000) or inductively coupled plasma-optical emission spectroscopy (Thermo Electron iCAP 6500 Duo) in the laboratory of Labtium Ltd. in Kuopio, Finland. The laboratories are accredited according to FINAS T025 (EN ISO IEC 17025). At both laboratories the analyses included blanks and duplicate analyses were carried out systematically for 5% of the samples. Bush leaves (GBW07602), poplar leaves (GBW07604), peach leaves (NIST 1547), tomato leaves (SRM 1573a), soil (NIST 2709, NIST 2710) and lake sediment (NW-WQB-1) were used as certified reference materials. This procedure allowed measurements of 34 elements.

### Data analysis

The animal-to-soil CRs were calculated using the equation

$$CR = C_{wo} /C_{soil} ,$$

where *C*_*wo*_ is the whole organism concentration of an element in a pooled sample of beetles or earthworms (mg kg^−1^ DW) and *C*_*soil*_ is the average concentration (mg kg^−1^ DW) of an element in a corresponding soil sample, collected to the depth of 10 cm. For beetles, the soil concentration used in calculations was the arithmetic mean of soil concentrations of all beetle sampling points of that year. The calculation of CRs was different for earthworms collected from the field; the arithmetic mean of soil concentrations of only those sampling points from which earthworms were pooled together was used as the soil concentration in CR calculation. Different approaches for calculation of soil concentration for beetles and earthworms were used because beetles can potentially move across all the sampling points, while earthworms are likely representatives of limited areas. Average concentrations and CRs were calculated for those elements for which less than five samples for earthworms and less than fifteen samples for beetles had a concentration below the detection limit of that element. In case a sample with a concentration below the detection limit was used for calculations, half of the detection limit was used as the concentration of that sample.

To investigate the correlation between field and mesocosm concentrations, scatterplots were produced, and linear regression analyses were performed. Similarly, scatterplots and linear regression were used for evaluating the relationship between concentrations observed in single beetle species (or genera) and in pooled multi-species beetle samples. In this analysis, genus-level (*Pterostichus* sp.) rather than species-level data was used for three species (*Pterostichus niger, Pterostichus melanarius* and *Pterostichus oblongopunctatus*), as samples of these species were pooled together for elemental analysis. These species are ecologically and morphologically similar (Jorum 1980; Symondson et al. 2000; Magura et al. 2008; Simon et al. 2016; Jowett et al. 2019). SPSS 27 for Windows (SPSS Inc., an IBM Company) was used for the statistical analyses.

## Results

The element concentrations in soil, earthworms and beetles are shown in Tables 2 and 3 together with the animal-to-soil CRs. In the field study, the concentrations and CR values of most elements were higher and in many cases an order of magnitude higher in the earthworms than in the ground beetles. Only the CR value of boron was clearly higher in the beetles than in the earthworms. Generally, the CR values of elements other than important nutrients (Ca, K, Na, P, S, Se, and Zn) were below one, indicating that uptake of these elements from soil into beetles and the earthworms is low. Cd was an exception to this as the CR values for both beetles and earthworms suggested possible accumulation.

**Table 2:** Geometric means (geometric standard deviations) of element concentrations (mg kg^−1^) in soil (S), (at the earthworm sampling points), earthworms (E) and earthworm-to-soil concentration ratios (CR) in mixed earthworm species collected from the field and in a mesocosm study with *Lumbricus terrestris*

Element	Field			Mesocosm		
	S (n = 9)	E (n = 9)	CR	S (n = 5)	E (n = 5)	CR
Ag	0.14 (1.49)	0.12 (1.85)	0.87 (2.58)	0.01 (1.00)	0.01 (1.00)	1.00 (1.00)
Al	7910 (1.18)	474 (1.88)	0.06 (1.83)	7201 (1.09)	1649 (1.32)	0.23 (1.31)
As	1.36 (1.53)	0.39 (1.77)	0.28 (1.86)	2.43 (1.05)	1.29 (1.16)	0.53 (1.14)
B	3.14 (1.32)	2.41 (1.41)	0.77 (1.47)	1.00 (1.95)	0.53 (1.03)	0.53 (1.96)
Ba	53.7 (1.46)	10.4 (1.36)	0.19 (1.61)	45.3 (1.09)	14.9 (1.58)	0.33 (1.49)
Be	0.21 (1.47)	< 0.1	n.a	0.10 (1.14)	0.03 (1.03)	0.26 (1.15)
Bi	< 0.1	< 0.1	n.a	0.05 (1.03)	0.05 (1.03)	1.02 (1.01)
Ca	3222 (1.21)	5800 (1.31)	1.81 (1.45)	3111 (1.16)	6292 (1.12)	2.02 (1.29)
Cd	0.09 (1.22)	3.52 (2.15)	40.0 (2.41)	0.09 (1.07)	4.11 (1.18)	40.5 (1.22)
Co	4.41 (1.28)	3.48 (1.75)	0.79 (1.91)	3.59 (1.06)	8.61 (1.21)	2.39 (1.20)
Cr	17.1 (1.19)	1.45 (1.73)	0.08 (1.84)	12.4 (1.11)	3.51 (1.34)	0.28 (1.29)
Cu	15.3 (1.89)	11.4 (1.56)	0.74 (2.05)	30.6 (1.08)	21.7 (1.06)	0.71 (1.12)
Fe	14,253 (1.34)	982 (1.81)	0.07 (1.98)	12,886 (1.08)	3036 (1.31)	0.23 (1.33)
K	1321 (1.34)	9106 (1.07)	6.89 (1.33)	1088 (1.09)	8661 (1.21)	7.96 (1.21)
Li	8.11 (1.34)	0.54 (2.06)	0.07 (2.31)	5.81 (1.23)	1.29 (1.89)	0.22 (1.56)
Mg	3358 (1.34)	1093 (1.29)	0.33 (1.33)	3645 (1.07)	1450 (1.15)	0.39 (1.12)
Mn	163 (1.47)	55.9 (1.47)	0.34 (1.43)	100 (1.11)	37.2 (1.29)	0.37 (1.28)
Mo	2.01 (2.71)	0.82 (1.74)	0.41 (2.54)	2.59 (1.31)	1.34 (1.11)	0.52 (1.35)
Na	138 (1.12)	3323 (1.11)	24.0 (1.16)	160 (1.25)	3618 (1.39)	22.7 (1.74)
Ni	10.9 (1.37)	2.31 (1.74)	0.21 (1.77)	11.1 (1.05)	4.34 (1.25)	0.39 (1.24)
P	659 (1.13)	10,075 (1.06)	15.3 (1.16)	880 (1.13)	9133 (1.15)	10.4 (1.21)
Pb	8.95 (1.84)	1.91 (2.35)	0.21 (3.87)	4.61 (1.09)	1.49 (1.36)	0.32 (1.28)
Rb	16.7 (1.31)	10.6 (1.21)	0.64 (1.36)	10.6 (1.18)	9.65 (1.12)	0.91 (1.16)
S	501 (1.71)	7918 (1.06)	15.8 (1.71)	558 (1.25)	7151 (1.15)	12.8 (1.36)
Sb	< 0.02	< 0.02	n.a	0.03 (1.03)	0.03 (1.03)	1.02 (1.01)
Se	< 0.5	1.45 (1.91)	n.a	0.58 (1.32)	3.89 (1.15)	6.77 (1.19)
Si	345 (1.05)	327 (1.34)	0.95 (1.32)	n.m	n.m	n.m
Sr	21.9 (1.13)	18.7 (1.26)	0.85 (1.28)	11.6 (1.29)	10.0 (1.16)	0.85 (1.35)
Th	2.44 (1.14)	0.14 (1.83)	0.06 (2.01)	1.69 (1.31)	0.43 (1.86)	0.26 (1.71)
Ti	1220 (1.11)	56.7 (2.04)	0.05 (1.89)	743 (1.29)	153 (1.29)	0.21 (1.33)
Tl	0.16 (1.81)	0.07 (1.39)	0.42 (1.84)	0.14 (1.36)	0.07 (1.51)	0.49 (1.15)
U	4.38 (4.84)	1.39 (7.31)	0.32 (3.29)	39.1 (1.23)	11.9 (1.26)	0.31 (1.54)
V	34.3 (1.42)	2.09 (2.26)	0.06 (2.58)	29.4 (1.10)	6.78 (1.36)	0.23 (1.34)
Zn	33.0 (1.37)	518 (1.71)	15.6 (2.04)	28.0 (1.09)	345 (1.21)	12.3 (1.32)

n.a. = value is not available because element concentration in earthworms or soil was below detection limit

n.m. = Si concentrations were not measured in the mesocosm study

**Table 3:** Geometric means (geometric standard deviations) of element concentrations (mg kg^−1^) in soil (S) at beetle sampling points, beetles (B) and beetle-to-soil concentration ratios (CR)

Element	S (n = 69)	B (n = 29)	CR
Ag	0.13 (1.53)	0.03 (1.39)	0.19 (1.39)
Al	6952 (1.45)	16.2 (1.77)	0.002 (1.77)
As	1.11 (1.41)	< 0.05	n.a
B	3.22 (1.39)	548 (1.48)	160 (1.49)
Ba	58.2 (1.55)	2.39 (1.55)	0.04 (1.55)
Be	0.18 (1.71)	< 0.1	n.a
Bi	< 0.1	< 0.1	n.a
Ca	3102 (1.45)	527 (1.22)	0.15 (1.22)
Cd	0.11 (1.78)	0.22 (1.97)	1.51 (1.97)
Co	4.00 (1.48)	0.14 (1.72)	0.03 (1.72)
Cr	14.7 (1.51)	< 0.5	n.a
Cu	15.5 (2.00)	13.6 (1.31)	0.63 (1.31)
Fe	11,758 (1.63)	61.7 (1.28)	0.005 (1.28)
K	1160 (1.41)	3727 (1.27)	3.02 (1.27)
Li	5.94 (1.96)	0.17 (1.46)	0.02 (1.46)
Mg	2683 (1.76)	727 (1.11)	0.24 (1.11)
Mn	180 (1.67)	43.7 (1.84)	0.21 (1.84)
Mo	1.46 (2.91)	0.85 (3.05)	0.25 (3.05)
Na	144 (1.35)	4366 (1.26)	29.1 (1.26)
Ni	10.4 (1.53)	0.55 (1.51)	0.05 (1.51)
P	660 (1.54)	4704 (1.12)	6.24 (1.12)
Pb	11.3 (1.89)	< 0.05	n.a
Rb	15.9 (1.47)	2.53 (1.41)	0.15 (1.41)
S	535 (2.09)	3187 (1.08)	4.39 (1.08)
Sb	< 0.02	< 0.02	n.a
Se	< 0.5	< 0.5	n.a
Si	344 (1.19)	59.1 (1.44)	0.17 (1.44)
Sr	22.5 (1.39)	2.59 (1.41)	0.11 (1.41)
Th	2.17 (1.42)	< 0.02	n.a
Ti	1070 (1.42)	1.46 (1.82)	0.001 (1.83)
Tl	0.15 (1.58)	0.01 (1.51)	0.06 (1.51)
U	2.64 (3.96)	0.01 (2.06)	0.001 (2.06)
V	31.5 (1.68)	< 0.1	n.a
Zn	32.4 (1.51)	101 (1.13)	2.81 (1.13)

Arithmetic means of the soil concentrations were used for calculation of the CR values

n.a. = value is not available because element concentration in beetles or soil was below detection limit

**Fig. 2:**
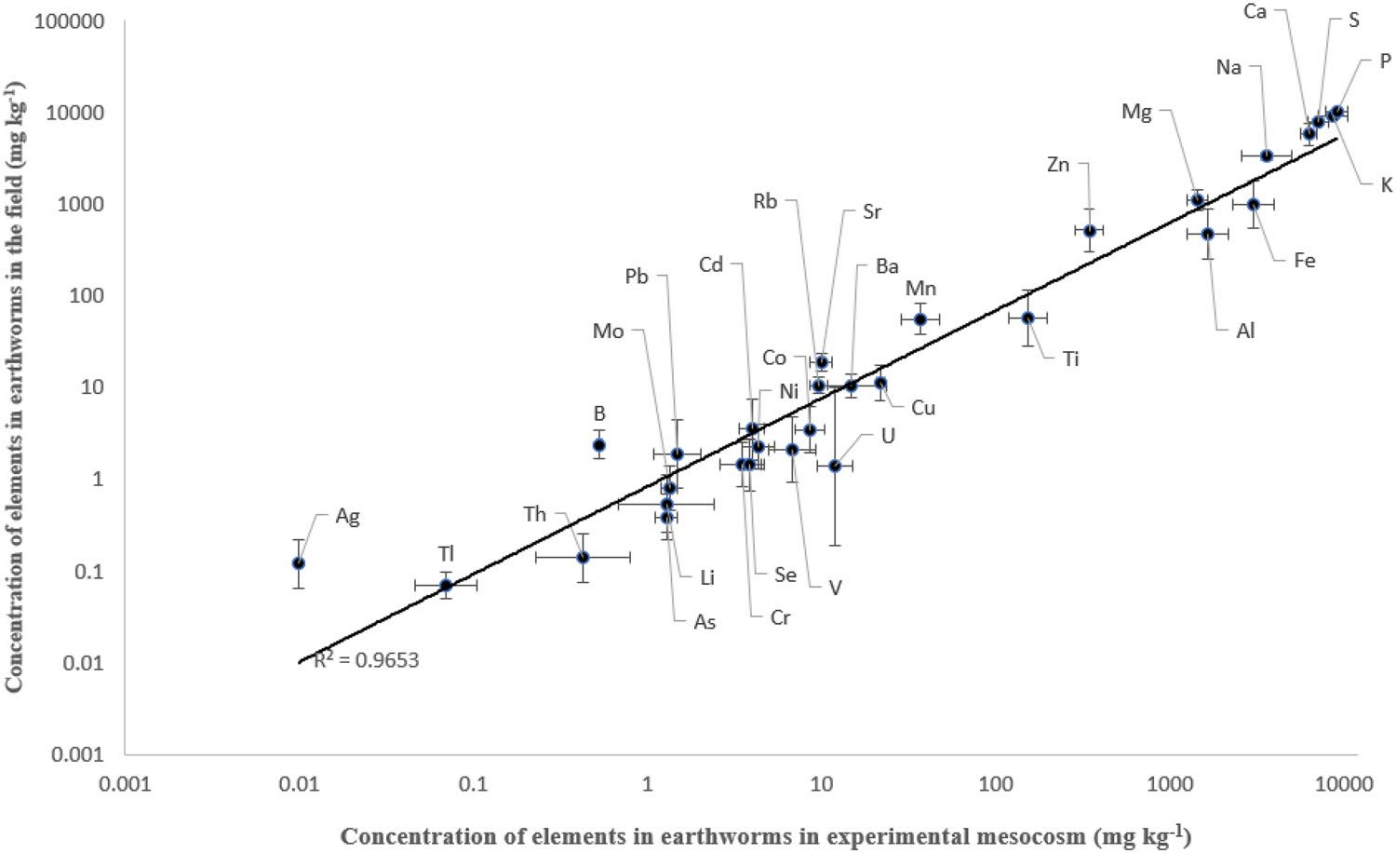
Concentrations of elements (mg kg^−1^) in earthworms collected from the field (n = 9) as a function of the concentrations of the elements observed in earthworms (n = 5) in an experimental mesocosm study (Tuovinen et al. 2016a). Solid line represents linear regression between the concentrations measured in the field and the mesocosm studies (Slope = 1.02; 95% confidence interval 0.96–1.08; R^2^ = 0.97). The values for Be, Bi and Sb are not shown as their concentrations in the field-collected animals were below the detection limit

For most of the elements, the concentrations in earthworms were quite similar in the field data and in the mesocosm experiment (Table 2 and Fig. 2). A difference higher than 10-fold was observed only for Ag, and a nearly 10-fold difference between the field and mesocosm data was observed also for U. The R^2^ value for the regression between field and mesocosm data was 0.97. The slope of the regression line was 1.02 (95% confidence interval 0.96–1.08), indicating no systematic over- or underestimation that might result from the use of experimental mesocosm data.

**Fig. 3:**
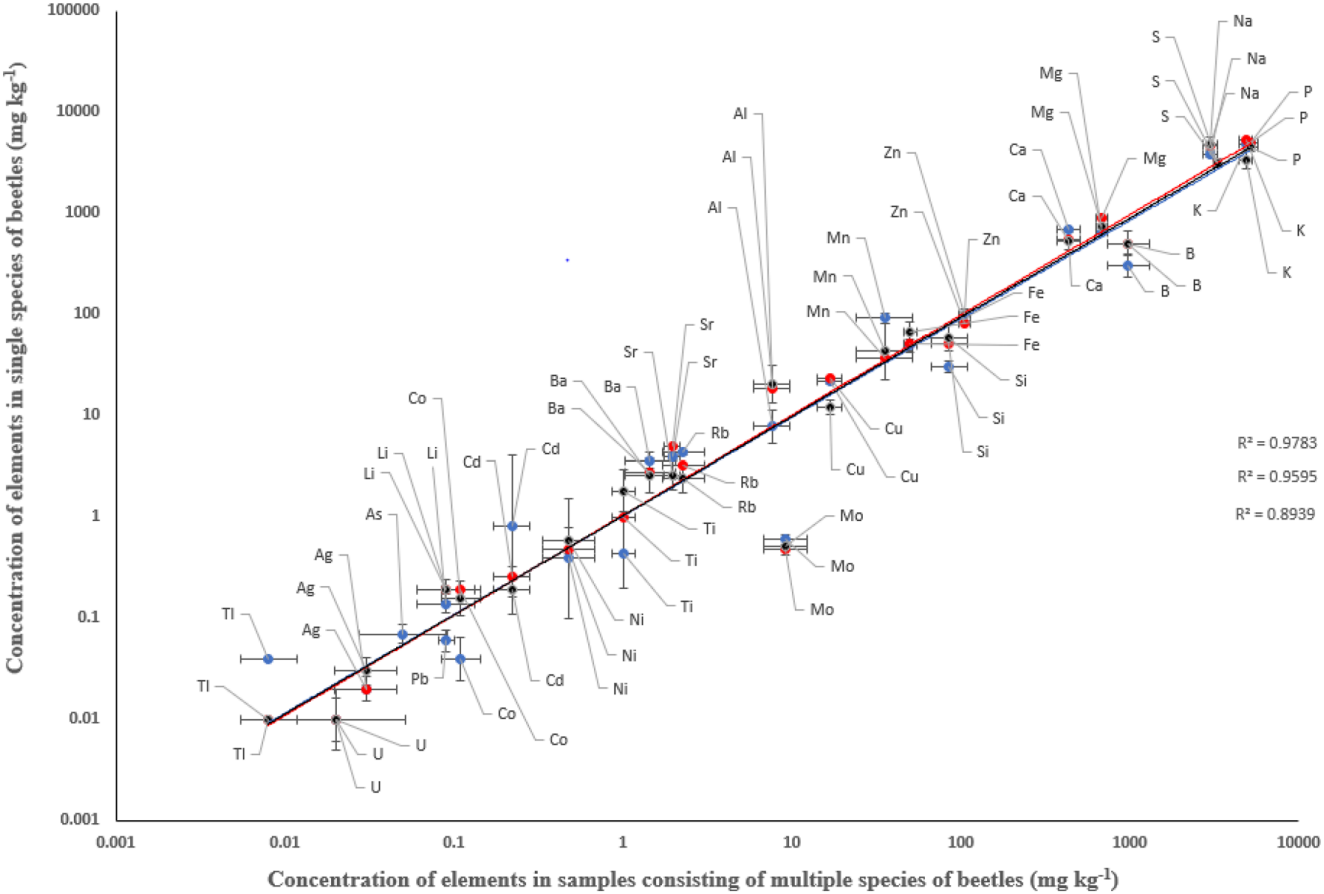
Concentrations of elements (mg kg^−1^) in *C. caraboides* (blue, n = 2)*, C. glabratus* (red, n = 1) and *Pterostichus sp.* (black, n = 21) in samples collected in 2007 as a function of the concentration of the elements measured in beetle samples (n = 5) consisting of several species, collected in 2008. Solid lines represent linear regression between the concentrations measured in individual species and the mixed samples. *C. caraboides*: slope = 1.00, 95% confidence interval 0.95–1.05; R^2^ = 0.98; *C. glabratus*: slope = 1.07, 95% confidence interval 0.99–1.15; R^2^ = 0.96. *Pterostichus sp*: slope = 0.91, 95% confidence interval 0.79–1.03; R^2^ = 0.89. The values for Be, Bi, Cr, Se, Th and V are not shown as their concentrations in the animals were below the detection limit

Also, the regression between species- or genus-specific concentrations in beetles (2007 data) and pooled multi-species data (all 2008 beetle data pooled) showed good fit, with R^2^ values of 0.89 for *Pterostichus* sp., 0.96 for *C. glabratus* and 0.98 for *C. caraboides* with slopes of 0.91 (95% CI 0.79–1.03), 1.07 (95% CI 0.99–1.15) and 1.00 (95% CI 0.95–1.05), respectively (Fig. 3). An obvious exception to the good fit was Mo. Its concentration in the multi-species data was more than an order of magnitude higher than in the single species or genera. The concentrations measured in beetle species, genera, or mixed species samples (corresponding to Fig. 3) are reported in Supplementary Table S1.

## Discussion

We investigated the element concentrations in beetles and earthworms and calculated CRs describing the transfer of 34 elements from soil to the animals. This information can be utilized when assessing risks of transfer of e.g., heavy metals and radionuclides to the environmental food webs in boreal forest ecosystems.

Among the elements studied, Cd was the only non-essential element that showed a CR value greater than one, indicating its accumulation in the animals studied. Similar observation was also made for red wood ants (genus *Formica*) at a former uranium mining site in Paukkajanvaara, Eastern Finland (Roivainen et al. 2022). Bioaccumulation of Cd into organisms, including earthworms, has been reported especially in acidic soils (Zhang and Reynolds 2019). Relatively low pH (4.5) was found also at the study site of the present study.

The CR values of many elements were an order of magnitude higher in earthworms than in beetles. For elements like U, the difference in CR values was even two orders of magnitude. A likely explanation for this finding is the fact that earthworms live in more intensive contact with soil and the transfer of elements can occur both via ingestion of soil and via direct skin contact with soil (Heikens et al. 2001). For beetles, the most important source is likely to be their food rather than contact with soil (Jelaska et al. 2014).

The use of CR values in radioecological models is based on the assumption that uptake of elements into organisms is linear and can therefore be described by a constant CR. Although this assumption may not be valid (Tuovinen et al. 2011, 2016a, b), CR-based modelling is widely used in radioecology. It is therefore of interest to compare the CR values found in this study to those published elsewhere. Only a few studies have reported CR values for transfer of elements from soil to beetles; the results of those studies are compared to our findings in Table 4. O'Quinn (2005) reported geometric means of beetle-to-soil CR values (dry weight-based) for Cd, Cu, Ni, U and Ti at a riparian contaminated area on Savannah River, previously used for producing nuclear materials. The CR values for Cu, and U for the beetles observed in the present study were of the same order of magnitude as those reported by O'Quinn. On the other hand, the results of the present study showed higher (≥ 5-fold) CR values for Cd and Ni and lower CR value for Ti than the values observed by O'Quinn. In a study by Bednarska et al. (2016), higher transfer of Cd and Ni based on dry weight was observed from soil to adult male of *P. oblongopunctatus* in a Scots pine forest from an unpolluted area in Southern Poland, compared to the CRs found in the present study. Arithmetic mean of bioaccumulation factors (dry weight-based) for several elements were reported in *H. rufipes* under field conditions in agricultural soil in southern Italy (Naccarato et al. 2020). The CR values of Ca, Fe, Rb, Sr and Zn in our study were within the ranges of the bioaccumulation factors observed at three different sites by Naccarato et al. However, the CR values in our study were lower for Cu and higher for Ba, Co, Li, Mg, Mn and Na than the ranges of corresponding values reported by Naccarato et al. In addition, the CR value observed by Naccarato et al. for U was fivefold higher than the CR value for U in our work. IAEA (2014) gives generic CR values for arthropods. As these CR values are calculated based on the fresh weight, they were converted to dry weight before comparison to our values. A conversion factor of 0.25 was used as given in IAEA (2014). The CRs for beetles found in this study are for many elements (Cd, Ni and Zn) within the ranges given for arthropods by IAEA. However, our CR value for Co is higher than the range reported by IAEA, while Sr and U showed lower CRs in our study than the values reported by IAEA.

**Table 4:** Comparison of concentration ratios observed in this study to those published elsewhere

Element	Present study	Other studies	IAEA generic values
Earthworms			
As	0.28	0.11–0.13^1^; 0.39–1.71^2^	0.35–4.64^a^
Cd	40.0	25.5–26.4^1^; 1.61–19.5^2^; 10.6–18.8^3^; 0.60^4^	2.29–123^a^
Cu	0.74	0.55–5.73^2^; 1.01–1.35^3^	
Fe	0.07	0.80^4^	
Mn	0.34		0.006–0.12^a^
Mo	0.41	0.42–2.21^2^	
Ni	0.21		0.04–2.84^a^
Pb	0.21	22.0–65.0^3^	0.01–16.4^a^
Se	6.77	23.5–271^2^; 1.09–1.49^5^	
U	0.32	0.08–0.31^6^; 0.09–0.52^7^; 0.22–0.42^8^; 0.09–0.25^9^	
Zn	15.6	2.40–2.70^1^; 2.90–19.3^2^; 1.15–1.75^3^; 0.77^4^	11.2–41.2^a^
Ground beetles			
Ba	0.04	0.002–0.006^10^	
Ca	0.15	0.04–0.48^10^	
Cd	1.51	0.26^11^; 20.8^12^	0.84–160^b^
Co	0.03	0.003–0.012^10^	0.01–0.02^b^
Cu	0.63	0.87^11^; 2.13–5.47^10^	
Fe	0.005	0.002–0.007^10^	
Li	0.02	0.0006–0.002^10^	
Mg	0.24	0.29–1.41^10^	
Mn	0.21	0.02–0.05^10^	
Na	29.1	0.12–0.20^10^	
Ni	0.05	0.01^11^; 15.0^12^	0.04–2.84^b^
Rb	0.15	0.05–0.33^10^	
Sr	0.11	0.03–0.12^10^	0.24–7.60^b^
Ti	0.001	1.70^11^	
U	0.001	0.01^10^; 0.004^11^	0.04–0.08^b^
Zn	2.81	1.17–3.67^10^	1.20–14.4^b^

^1^Nannoni et al. (2011), ^2^Chen et al. (2014), ^3^Wang et al. (2018), ^4^Latifi et al. (2020), ^5^Yue et al. (2021), ^6^Oliver et al. (2008), ^7^Giovanetti et al. (2010), ^8^Lourenço et al. (2011), ^9^Mrdakovic Popic et al. (2012), ^10^Naccarato et al. (2020), ^11^O'Quinn (2005), ^12^Bednarska et al. (2016)

^a^Generic values for annelids (IAEA 2014)

^b^Generic values for arthropods (IAEA 2014)

There are several studies reporting the transfer of elements from soil to different earthworm species (Table 4). The CR for U in earthworms in our study is within the wide variety of CR values found in previous studies (Oliver et al. 2008; Giovanetti et al. 2010; Lourenço et al. 2011; Mrdakovic Popic et al. 2012). The values reported by Giovanetti et al. (2010) were based on the fresh weight and were therefore converted to dry weight by a conversion factor of 0.21, as suggested by the authors, before comparison to our data. The values reported by Chen et al. (2014) showed variation of CRs for As, Cd, Cu, Mo, Se and Zn at four different sites in the United States. Our findings were within these ranges except for Cd, which was higher and Se, which was lower in our study. In the study by Wang et al. (2018), transfer of heavy metals into earthworms was lower than in our study for Cd and Zn, whereas higher transfer of Cu and Pb was observed. A study in Kosovo (Latifi et al. 2020) showed lower CR values in earthworms from five different sites for Cd and Zn but higher CRs for Fe than those observed in our study. The CR values reported by Nannoni et al. (2011) for As, Cd, and Zn were lower than the values in our study in two earthworm species, *A. rosea* and *N. caliginosus*, collected from a smelter contaminated area in northern Kosovo. Yue et al. (2021) reported lower transfer of Se in earthworms exposed to artificial soil in comparison to the CR value found in our mesocosm experiment. Comparison of our data with generic CRs for annelids after converting the fresh weight-based values by a conversion factor of 0.17 (IAEA 2014), showed that our CRs for many elements (As, Cd, Ni, Pb and Zn) are within the ranges of CR values reported for annelids by IAEA. However, higher transfer of Mn was observed in our study than suggested by the range of generic values given by IAEA for Mn. Overall, the high variation of CRs in these studies is consistent with findings indicating that CRs are highly site-specific and affected by factors such as concentration of the studied element in soil (Tuovinen et al. 2016a) as well as concentrations of other elements and soil properties (Roivainen et al. 2011a, b). As all influential factors are typically not known for the site for which predictions are produced, CR-based prediction models are inherently uncertain, heavily influenced by the selection of CR values and able to produce only approximate predictions.

A problem related to field studies is that organisms move, and it is difficult to define the representative soil concentration when calculating the CRs in field studies (Mrdakovic Popic et al. 2012). However, field studies are needed in addition to controlled laboratory experiments to give a more realistic picture of the transfer (Mrdakovic Popic et al. 2012). Here, we showed that the results obtained for earthworms in a field study were generally comparable to the corresponding results obtained in a controlled experimental system using the same soil. Similar results were obtained when soil-to-plant transfer data from field studies (Roivainen et al. 2011a, b) was compared to the data from the mesocosm study (Tuovinen et al. 2016a). The mesocosm study design used by Tuovinen et al. (2016a) seems to adequately reflect the transfer of elements in natural habitat. This is an important finding and supports the usefulness of experimental approaches in studying transfer of elements into organisms. Further comparisons of experimental and field studies would be highly valuable.

Another important result was the finding that the concentrations in specific taxa of ground beetles were similar to those measured in pooled samples including several species, despite some differences in ecology and diets of the species studied. *C. caraboides* can be found in damp woodlands and typically feed on snails (Ingerson-Mahar 2002). *C. glabratus* is also found in damp environments in woodlands as well as in other habitats, including peat bogs and old coniferous forests (Vainikainen et al. 1998; Filippov 2006). Snails and earthworms form the main diet of these animals (Jelaska et al. 2007; Sota and Nagata 2008; Ikeda et al. 2012; Simon et al. 2016). *Pterostichus sp*. are often abundant in woodlands, grasslands, and spruce forests: they are scavengers and predators, feeding on wide range of small insects and larvae (Symondson et al. 2000; Magura et al. 2008; Simon et al. 2016; Jowett et al. 2019). Similarity of element concentrations in these beetle species supports the use of generic model parameters (such as CR values) for a group of related species, an approach widely used in radioecological modelling due to the sparsity of species-specific data (IAEA 2014).

It should be noted that also the earthworm data of the present study can be considered to support the use of generic model parameters, as similar concentrations were observed for the single anecic species (*Lumbricus terrestris*) used in the mesocosm experiment and for the pooled samples collected from the field, consisting of several earthworm species representing different ecological groups, including anecic (*Lumbricus terrestris*), endogeic (*Aporrectodea caliginosa*) and epigeic (*Dendrobaena octaedra*, *Lumbricus rubellus* and *Lumbricus castaneus*) species.

It is important to note that all ground beetle samples were collected from the same site. Thus, comparison of single beetle species (or genus) to pooled samples was possible without confounding factors that might result from different environmental conditions. Similarly confounding factors related to different soil properties was avoided in the comparison of the mesocosm and field data, as soil from the field study site was used in the mesocosm. Avoidance of confounding factors is an obvious strength of the study. However, this strength is associated with a limitation: the study did not produce any data about how transfer of elements into organisms is affected by soil properties or other environmental variables. Small number of samples was another limitation particularly in the comparison of taxon-specific and multispecies beetle samples, as only one or two samples per species were available for two species.

## Conclusion

To increase understanding of the transfer of elements into organisms and to provide data needed in models used for assessing environmental risks, concentrations of 34 elements and corresponding animal-to-soil CR values were reported for ground beetles and earthworms collected from a boreal forest site. Concentrations found in wild earthworms were highly correlated with those measured in a mesocosm experiment using the soil collected from the field site, indicating that results from mesocosm studies can be used for predicting transfer of elements in natural conditions. Furthermore, concentrations in single beetle or earthworm species (or single genus in case of *Pterostichus* sp.) were found to be similar to those observed in samples consisting of several related species. This finding supports the use of generic model parameters for a group of related species, an approach commonly used in current radioecological models due to the sparsity of species-specific data. Approximate model prediction is better than no prediction.

## Supplementary Information

Below is the link to the electronic supplementary material.

Supplementary file1 (DOCX 19 KB)

## Data Availability

All data generated or analysed during this study are included in this published article [and its supplementary information files].
