# Supplementary material for: Transfer of elements from soil to earthworms and ground beetles in boreal forest
Source: Radiat Environ Biophys. 2023 Apr 17;62(3):403–14. doi: 10.1007/s00411-023-01027-2 (PMC10356874; doi:10.1007/s00411-023-01027-2)
Supplement: Supplementary file 1 — Supplementary file1 (DOCX 19 KB) [file 411_2023_1027_MOESM1_ESM.docx]

**Transfer of elements from soil to earthworms and ground beetles in boreal forest**

Soroush Majlesi^a^, Päivi Roivainen^a^, Anne Kasurinen^a^, Tiina Tuovinen^a^, Jukka Juutilainen^a^

^a^University of Eastern Finland, Department of Environmental and Biological Sciences, P.O. Box 1627, 70211 Kuopio, Finland

Corresponding author Soroush Majlesi

University of Eastern Finland, Department of Environmental and Biological Sciences

P.O. Box 1627, 70211 Kuopio, Finland

email soroush.majlesi@uef.fi

Tel +358403553199

ORCID ID 0000-0003-1509-644X

Table S1. Geometric means (geometric standard deviations) of element concentrations in soil (mg kg^-1^) at beetle sampling points (S), three individual species of beetles (*C. caraboides, C. glabratus* and *Pterostichus sp.*), (mg kg^-1^) from 2007 and group of species, collected in 2008.

| Element/species | S (n = 69) | *C.caraboides* (n=2) | **C. glabratus* (n=1) | | *Pterostichus sp.* (n=21) | Group of species (n=5) |
| --- | --- | --- | --- | --- | --- | --- |
| Ag | 0.13 (1.53) | 0.02 (1.33) | 0.02 | 0.03 (1.36) | | 0.03 (1.53) |
| Al | 15.9 (1.47) | 7.87 (1.46) | 18.3 | 20.7 (1.53) | | 7.63 (1.28) |
| As | 6952 (1.45) | 0.07 (1.23) | <0.05 | <0.05 | | 0.05 (1.82) |
| B | 1.11 (1.41) | 306 (1.31) | 500 | 506 (1.31) | | 982 (1.33) |
| Ba | 3.22 (1.39) | 3.64 (1.19) | 2.77 | 2.57 (1.49) | | 1.45 (1.42) |
| Be | 58.2 (1.55) | <0.1 | <0.1 | <0.1 | | <0.1 |
| Bi | 0.18 (1.71) | <0.1 | <0.1 | <0.1 | | <0.1 |
| Ca | 535 (2.09) | 687 (1.04) | 551 | 535 (1.21) | | 438 (1.17) |
| Cd | <0.1 | 0.81 (5.00) | 0.26 | 0.19 (1.73) | | 0.22 (1.28) |
| Co | 3102 (1.45) | 0.04 (1.63) | 0.19 | 0.16 (1.48) | | 0.11 (1.31) |
| Cr | 0.11 (1.78) | <0.5 | <0.5 | <0.5 | | <0.5 |
| Cu | 4.00 (1.48) | 21.9 (1.07) | 23.4 | 12.1 (1.18) | | 16.8 (1.18) |
| Fe | <0.02 | 49.1 (1.15) | 52.0 | 66.8 (1.27) | | 50.1 (1.09) |
| K | <0.5 | 4788 (1.08) | 5310 | 3358 (1.21) | | 4863 (1.09) |
| Li | 14.7 (1.51) | 0.14 (1.23) | 0.19 | 0.19 (1.29) | | 0.09 (1.48) |
| Mg | 344 (1.19) | 654 (1.02) | 905 | 736 (1.09) | | 685 (1.08) |
| Mn | 22.5 (1.39) | 92.4 (1.12) | 37.4 | 43.2 (1.91) | | 35.3 (1.48) |
| Mo | 15.5 (2.00) | 0.60 (1.11) | 0.49 | 0.51 (1.19) | | 9.09 (1.34) |
| Na | 2.17 (1.42) | 3848 (1.09) | 4630 | 4821 (1.16) | | 2994 (1.10) |
| Ni | 11758 (1.63) | 0.39 (3.88) | 0.48 | 0.59 (1.36) | | 0.48 (1.43) |
| P | 1070 (1.42) | 5008 (1.03) | 5030 | 4544 (1.11) | | 5234 (1.09) |
| Pb | 1160 (1.41) | 0.06 (1.27) | <0.05 | <0.05 | | 0.09 (1.11) |
| Rb | 5.94 (1.96) | 4.33 | 3.27 | 2.44 (1.39) | | 2.27 (1.33) |
| S | 0.15 (1.58) | 3153 (1.04) | 3140 | 3170 (1.08) | | 3280 (1.05) |
| Sb | 2683 (1.76) | 0.02 (1.06) | <0.02 | <0.02 | | 0.03 (1.42) |
| Se | 180 (1.67) | <0.5 | <0.5 | <0.5 | | <0.5 |
| Si | 2.64 (3.96) | 30.1 (1.14) | 50.8 | 58.1 (1.35) | | 85.5 (1.28) |
| Sr | 1.46 (2.91) | 3.96 (1.07) | 4.95 | 2.57 (1.38) | | 1.96 (1.11) |
| Th | 144 (1.35) | <0.02 | <0.02 | <0.02 | | <0.02 |
| Ti | 31.5 (1.68) | 0.44 (2.24) | 1.01 | 1.81 (1.62) | | 1.01 (1.18) |
| Tl | 10.4 (1.53) | 0.04 (1.00) | 0.01 | 0.01 (1.00) | | 0.008 (1.46) |
| U | 660 (1.54) | 0.01 (1.63) | 0.01 | 0.01 (1.98) | | 0.02 (2.56) |
| V | 11.3 (1.89) | <0.1 | <0.1 | <0.1 | | <0.1 |
| Zn | 32.4 (1.51) | 101 (1.04) | 82.7 | 101 (1.14) | | 105 (1.08) |

* No geometric means or geometric standard deviations were given for *C. glabratus* since only one sample was measured.
